# A transdiagnostic approach to investigate of the relationships between anxiety sensitivity and health anxiety: the mediated roles of distress tolerance and emotion regulation

**DOI:** 10.3389/fpsyt.2025.1478442

**Published:** 2025-02-05

**Authors:** Hamid Mohsenabadi, Mohammadreza Pirmoradi, Komeil Zahedi Tajrishi, Banafsheh Gharraee

**Affiliations:** Department of Clinical Psychology, School of Behavioral Sciences and Mental Health (Tehran Institute of Psychiatry), Iran University of Medical Sciences, Tehran, Iran

**Keywords:** anxiety sensitivity, health anxiety, distress tolerance, emotion regulation, transdiagnostic approach

## Abstract

**Background:**

Emotion regulation (ER) and distress tolerance (DT) are considered transdiagnostic risk factors for a range of anxiety disorders. This study investigated the relationship between anxiety sensitivity (AS) and health anxiety (HA) in the general population, focusing on the mediating roles of DT and ER.

**Methods:**

The study was conducted as a cross-sectional survey from October to December 2023 in Tehran Province, Iran. A total of 971 individuals participated in this study (52.8% female; mean age 39.04 years, SD=10.64). Participants completed self-report questionnaires to assess HA (The Short Health Anxiety Inventory), AS (The Anxiety Sensitivity Index-3), DT (The 15-item Distress Tolerance Scale) and ER (The 10-item Emotion Regulation Questionnaire). We used structural equation modeling (SEM) to examine the hypothesis that DT and ER would mediate the relationship between AS and the HA.

**Results:**

AS was modestly related to all measures (r from -0.40 to 0.55). According to the SEM analysis, AS (β = 0.45, 95%CI [0.34, 0.56]) had a significant direct effect on HA. However, the analysis of the indirect effects revealed that both DT (β = 0.10, 95% CI [0.06, 0.16]) and emotion regulation strategies—cognitive reappraisal (β = 0.06, 95% CI [0.01, 0.11]) and expressive suppression (β = 0.11, 95% CI [0.06, 0.18])—act as partial mediators in the relationship between AS and HA.

**Conclusions:**

AS plays a crucial role in predisposing individuals to HA. The mediating roles of DT and ER provide insight into the link between AS and HA. Nevertheless, the study’s cross-sectional design and reliance on a non-clinical sample limit the applicability of the results. Further research involving different samples and intervention studies is needed to validate and expand upon these findings.

## 1 Introduction

### 1.1 Health anxiety

Health anxiety (HA), alike known as illness anxiety, is a common issue, impacting up to 6% of general individuals at some point in their lives and up to twenty percent of medical outpatients (1). Excessive worry about one’s health and fear of getting sick are key features of HA, leading to constant monitoring of symptoms and unproductive facts seeking and safety measures (1). HA, while mild forms can encourage beneficial behaviors such as attending preventive medical check-ups, becomes a disabling condition when excessive. Severe health anxiety leads to significant distress, challenging patient-doctor relationships (2), increased healthcare utilization (3), and a considerable economic burden (4). It affects approximately 3.4% of the adult population (5) and is strongly associated with psychological distress and functional impairment (3, 5). As a serious and costly public health issue, untreated HA often becomes chronic (6), resulting in enduring consequences for individuals, healthcare systems, and society (7). The effect of HA on one’s health and the use of medical services can substantially elevate healthcare costs. In a natural longitudinal examination, it was revealed that individuals suffering from severe HA incurred 41–78% more healthcare expenses annually (2).

Numerous examinations have concentrated on the cognitive and emotional factors connected with a greater probability of experiencing excessive HA (8–10). In several studies (11–14), it has been discussed, investigated, and demonstrated that anxiety sensitivity (AS), emotion regulation (ER) and distress tolerance (DT) which are known as three transdiagnostic factors are linked to HA. These factors provide valuable insight into the underlying mechanisms of HA, offering potential targets for therapeutic interventions.

### 1.2 Health anxiety and anxiety sensitivity

Anxiety sensitivity (AS) as transdiagnostic risk factor for psychiatric disorders refers to the negative emotional reactions to anxiety-related senses or symptoms. It involves the fear of bodily senses related to physiological arousal and the belief that these senses may result in negative physical, social, or psychological impacts (15, 16). AS is a result of dysfunctional beliefs and can lead to intense anxiety-related fear of physical sensations. Several studies highlight the connection between AS and illness anxiety disorder. As part of the cycle of health anxiety, AS can intensify an individual’s tendency to be overly vigilant about bodily senses and to experience distress when they occur (17, 18). AS has been observed to be linked with HA in both non-clinical and clinical adult samples. Research has found that greater levels of AS relates to greater HA (11, 19). In contrast, the absence of a significant prospective relationship between AS and HA is incompatible with some earlier work (12).

### 1.3 Health anxiety and distress tolerance

Distress tolerance (DT) represents the capacity to endure unfavorable psychological and physical states (20). It influences how distressing stimuli are appraised and the perceived capability to manage them (21). DT measures a person’s capability to endure uncomfortable emotional states and assesses their ability to handle distress (22). The behavioral index of DT measures the mastery to handle a challenging situation or task while striving toward a goal in front of negative feelings (20).

Studies indicate that clinically meaningful anxiety levels are related to more down levels of DT (23). According to a study (24), these results imply that DT may be an essential component in the development of anxiety disorders in overall. Persistent anxiety symptoms may result from actions linked to low DT (25). Studies suggest that DT and HA are negatively associated in a wide variety of nonclinical individuals (25, 26). Individuals with low DT are driven to flee from interior experiences, which exacerbates anxiety, since they believe they are unable to tolerate bad internal states, such as by believing that illness-related beliefs are dangerous and uncontrollable (25, 26). The concept of DT has obtained little consideration in the literature on HA despite several studies highlighting its significant role in general anxiety (26–29).

### 1.4 Health anxiety and emotion regulation

Emotional regulation (ER) involves trying to alter emotions by either evoking or sustaining emotional experiences, or by adjusting and effectively manage their frequency, strength, or duration (30). Our theoretical framework is anchored in Gross’s (2015) process model of ER, which stands as the most commonly utilized model of ER within the field of affective science. This model introduces a framework for emotion regulation that categorizes strategies into five distinct groups: the selection of specific scenarios, the adjustment of those scenarios, the way attention is focused, the reappraisal of cognitive perceptions, and the modification of responses (31).

Within the Gross’s model, cognitive reappraisal (CR) and expressive suppression (ES) serve as primary illustrations of strategies for regulating emotions. CR, which focuses on changing cognitive antecedents, requires a reinterpretation or intentional alteration in the perception of an emotional event or stimulus, aiming for a more balanced or positive perspective. Conversely, ES targets the modulation of emotional responses by restraining the outward expression of emotions, essentially inhibiting any reaction during the last phase of the ER process (31). In the field of ER, CR is often considered an adaptive strategy and ES considered a maladaptive strategy. Utilizing CR regularly is correlated with numerous beneficial long-term effects, such as enhanced overall well-being and social functional (32). On the other hand, excessive reliance on ES generally correlates with suboptimal outcomes, including diminished social functional and well-being (33). Certainly, mental health conditions like depression and anxiety disorders are often typified by a decreased reliance on CR and an increased reliance on ES (34, 35). The number of research exploring the contribution of and/or association between HA and ER In adults. ER difficulties have been positively correlated with HA (36, 37). Studies show that unhealthy emotional regulation techniques like suppression and catastrophizing are connected to HA (38, 39). Also (40), discovered that ER served as a mitigating factor for HA, but its impact was not direct. Exploring the relationship between ER and HA is valuable because the findings could enhance our understanding of the origins of HA, potentially leading to more effective, evidence-based interventions and assessments.

### 1.5 Anxiety sensitivity, distress tolerance and emotion regulation

An important transdiagnostic factor is anxiety sensitivity (AS), which involves the fear of physiological arousal senses and the view that they could lead to negative outcomes (16). Another related transdiagnostic factor is difficulties in ER, which involves an individual’s capacity to detect, process, and reply to emotions (33, 41). Previous study in adults supports a connection between ER and AS over time (42). Prior research has found significant interacting effects despite the fact that AS and ER are distinctive transdiagnostic variables (43, 44). In fact, it has been demonstrated that AS and ER are connected to the emergence, maintenance, and management of anxiety-associated pathologies and symptoms (43–45). For instance, prior research indicates that reduced ER capacity may act to intensify AS, which in turn may impact a variety of other anxiety consequences such as worry and panic (43). Current empirical research and established theoretical models indicate that an individual’s ER capacity may have a role in the development of AS (44). On the other hand, elevated AS levels may theoretically make it more difficult for a person to use ER techniques that are necessary to reduce symptoms. Previous research in clinical populations demonstrates that modifications in ER and AS mediate abatement in anxiety symptoms in individuals with anxiety-related illnesses (e.g., social phobia, PTSD, and generalized anxiety disorder (45);. All of these findings point to the importance of ER in transdiagnostic anxiety processes, including sensitivity to anxiety and signs correlated with anxiety (41). However, it is not possible to interpret these effects as causative due to the cross-sectional character of these investigations.

The capacity of a person to withstand unfavorable emotional condition (DT) and their inclination to evaluate their own feelings of anxiety as detrimental (AS) are conceptually similar and yet can be considered as separate constructs. Schmidt et al. (2011) investigated the correlation between AS and DT, highlighting that DT involves a general capacity for enduring emotional distress, while AS pertains specifically to the endurance of anxiety and its associated feelings (41). They noted that DT encompasses a wide array of negative emotional experiences, but AS distinctly targets managing anxiety, suggesting the possibility that AS might act as an element within the broader trait of DT (22, 46). Accordingly, if individuals have lower grades of DT, they may be more likely to experience anxiety issues due to their reduced capacity to handle and endure negative emotional states, leading them to avoid or escape situations that provoke anxiety (28).

When DT and AS have been studied together, conflicting findings arise regarding their distinct connections with symptoms of anxiety. Some research shows that AS is strongly connected to anxiety symptoms, whereas DT is not (47), but alternate research suggests that DT is unusually associated with anxiety symptoms, whereas AS is not (24, 48). Differences in previous studies, findings are likely attributable to a variety of agents including disparities in the demographics of participants, such as their age and symptom severity, as well as methodological variations like the duration of the study and the methodologies applied (e.g., measures used).

Anxiety and AS are inversely correlated with DT (28, 49, 50). In both nonclinical and treatment-seeking populations, a correlation has been established between DT and AS (49, 51). Despite documented negative relationships between DT and AS, to our knowledge, no studies have clearly examined the associations among AS, DT, and symptoms of HA in one combined model

### 1.6 Distress tolerance and emotion regulation

The research suggests that DT likely influences the choice of specific ER strategies an individual uses. In emotion research, high DT aids adaptive emotive reactions to life tension by serving as an ER skill. It enables individuals to select and implement effective strategies during challenging situations (52, 53). To express it another way, DT serves as a broad factor relating to individual differences that facilitates particular behaviors for regulating emotions. This viewpoint suggests that people with high DT are probable to engage in positive ER practices such as problem-solving and labeling emotions when faced with stress, while those with low DT may resort to less effective ER techniques like suppression and avoidance (21, 54). Linking adaptive ER behaviors might provide a reason for the relationship between DT and health outcomes (55). Quantitative study conclusions suggest that there are meta-analytic relationship between DT and various ER strategies. The investigation revealed that high DT showed moderate connections with maladaptive ER methods like rumination, worry, experiential avoidance and expressive suppression, with correlations ranging between -.19 and -.57. Conversely, DT had relatively weaker positive associations with effective ER including reappraisal, mindfulness and problem-solving, with correlations ranging between.08 and.38 (52). Previous investigation on DT in healthy individuals has also demonstrated that higher DT is linked to adaptive ER, encompassing impulse control and emotional awareness (56, 57).

### 1.7 Summary

The problem of HA is significant in clinical settings and requires further clarification. AS is a crucial factor that predisposes individuals to HA. AS has been identified in early studies as a significant risk factor for HA (58), playing a predictive role in this study.

While previous research has explored the relationships between AS and HA, a critical gap remains in understanding the *mechanisms* that explain this link. Specifically, few studies have examined the mediating roles of DT and ER, two constructs that are central transdiagnostic factors of emotional disorders. Our study is among the first to integrate these variables into a comprehensive model, highlighting their mediating roles in the AS-HA relationship. Additionally, understanding these mediators is crucial for developing targeted interventions. Unlike earlier studies that primarily focused on direct relationships, this research provides insights into *how* and *why* AS translates into HA, offering practical applications for clinical settings.

**Figure 1:**
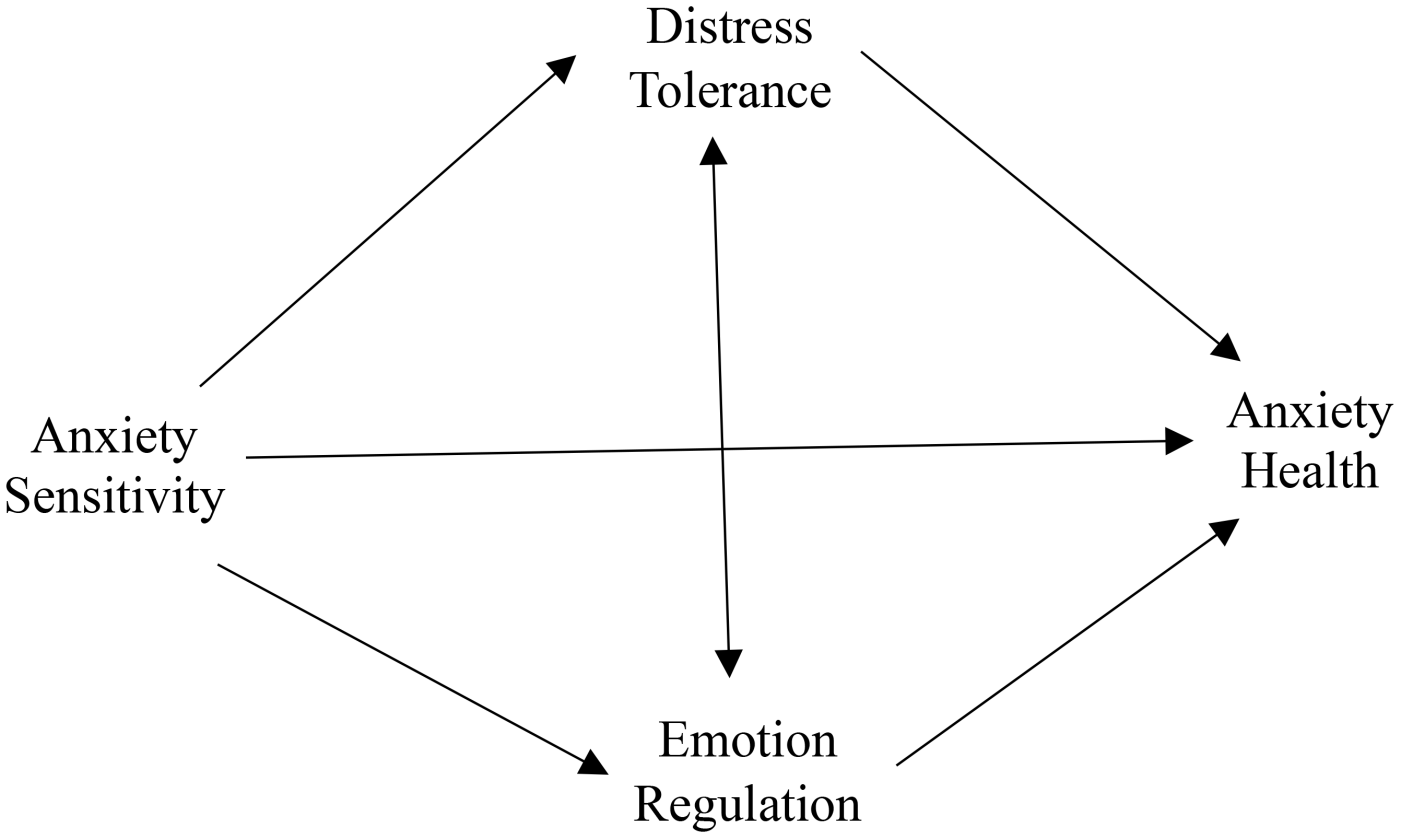
Initial hypothetical model of the relationship between anxiety sensitivity and anxiety health.

As mentioned earlier, both HA and AS are associated with abnormal processing of ER and DT, AS may decrease people’s DT and then cause HA symptoms. While ER and DT have each been separately associated with various anxiety outcomes, the current work explicitly models both factors as potential mediators. This dual-mediation design offers a more comprehensive view of the underlying mechanisms at play, thereby illuminating practical intervention targets. Therefore, the primary purpose of the study was to specify the role of ER and DT in the association between AS and HA (see **Figure 1**). We conceptualized AS as comprising of three dimensions: (1) physical concerns, (2) social concerns and (3) cognitive concerns, while HA was analyzed in terms of two dimensions: illness likelihood and negative consequences of illness. ER was defined with two dimensions: cognitive reappraisal (CR) and expressive suppression (ES), while DT was investigated in four dimensions: regulation, tolerance, appraisal, and absorption. Based on existing publications and previous results, we developed several assumptions. Prior research has indicated a link between AS and HA. Consequently, we hypothesized a positive relationship between AS and HA. Additionally, we proposed that DT, ES, and CR would act as mediators between AS and HA.

## 2 Method

### 2.1 Participants

This study used a convenience sample of 971 participants. The Participants were from Tehran city located in Iran country. The age group of the participants varied between 18 and 65 (M_Total_ = 39.04, SD_Total_ = 10.64). 52.8% were female (513 females; *Mean* age = 38.63, *SD* = 10.80) and 47.2% were male (458 males; *Mean* age = 39.49, *SD* = 10.44). The educational background of the participants varied: 3.7% (n=36) had some undergraduate education, 21.1% (n=205) held a diploma, 39.1% (n=380) had earned a bachelor’s degree, 30.4% (n=295) had obtained a master’s degree, and 5.7% (n=55) had achieved a doctoral degree. Concerning employment status, 57.8% (n=561) were employed, 26.9% (n=261) were student, 10.9% (n=106) were unemployed, 3% (n=29) were retired and 1.4% had lost their jobs. Fifty-nine percent of the sample reported their marital status as married (n=573), 41% as Single/divorced/windowed (n=398).

### 2.2 Procedure

The current study acquired approval from the Association for Ethics at the Iran University of Medical Sciences. Various social media platforms were utilized to recruit participants, from October to December 2023. Advertisements were placed on digital applications (such as WhatsApp, Instagram, Telegram) to invite participation in the investigation. A web link, which was provided in these advertisements, contained information about the study’s objective on its first page. By approving to participate and confirming the consent form, participants were then given access to do the surveys, submit demographic details, and receive a set of questionnaires. Participants must spend a minimum of 20 minutes completing questionnaires to ensure data quality. Participation required individuals to be a minimum of 18 years old. Participation was voluntary, with no compensation offered; moreover, participants were ensured confidentiality due to the unidentified essence of the questionnaire completion process.

Inclusion criteria comprised: (1) aged 18 to 65, (2) people who volunteered to take part in the study and (3) people who were able to done questionnaires on their own. Exclusion criteria included (1) inadequate proficiency in Persian and (2) no access to the Internet.

### 2.3 Measures

#### 2.3.1 Health anxiety

The Short Health Anxiety Inventory (SHAI) is a tool used to consider health-related anxiety via 18 items. Over time, various factor constructions have been presented for the SHAI, but this study employed a two-factor model. The two factors used in this study are “health anxiety,” which comprises 14 items, and “negative consequences of illness,” which includes 4 items. Participants select from four response options per item to reflect their feelings over the past six months. Previous studies have indicated the SHAI to have high internal consistency (59). The Persian version (60) has demonstrated satisfactory internal consistency (α = .88 for overall scale), temporal stability (r = .7) and construct validity. In the current study, the SHAI demonstrated excellent internal consistency (α = .80).

#### 2.3.2 Anxiety sensitivity

The Anxiety Sensitivity Index-3 (ASI-3) is a self-report questionnaire designed to assess sensitivity to anxiety symptoms across three domains: physiological, cognitive, and social. It contains 18 items that participants rate on a 5-point Likert scale ranging from 0 (Not at All) to 4 (Very Much). The ASI-3 provides both a total score and three subscale scores, with each subscale consisting of 6 items. Items are scored based on the frequency and severity of these symptoms, with total scores ranging from 0 to 72. Higher scores reflect greater sensitivity to anxiety (61). Research on the Persian versions of the ASI-3 has confirmed that both the total score and the subscales exhibit high internal consistency (with a Cronbach’s alpha of 0.93) and show strong convergent and divergent validity (19). In the current study, the ASI-3 indicated excellent reliability, with a Cronbach’s alpha of 0.94 for the total scale, and alpha values of 0.86 for the physiological, 0.85 for the cognitive, and 0.80 for the social subscales.

#### 2.3.3 Distress tolerance

Simons & Gaher (2005) developed The Distress Tolerance Scale *(*DTS), a five-point Likert scale with 15 items ranging from 1 (Strongly Disagree) to 5 (Strongly Agree). Using confirmation factor analysis, it has been found that there is a higher-order General DTS factor that consists of all items and corresponds to the total score and that there are four lower-order factor scales: Appraisal (6 items); Tolerance (3 items); Absorption (3 items); and Regulation (3 items) (17). As part of the primary validation study, the DTS had high internal consistency in a college sample (Cronbach’s alpha = 0.89), good divergent, criterion, and convergent validity. In the current study for the DTS, the Cronbach alphas were: Total Scale = 0.87; Appraisal = 0.80; Absorption = 0.73; Tolerance = 0.78; and Regulation = 0.78.

#### 2.3.4 Emotion regulation

The Emotion Regulation Questionnaire (ERQ), developed by Gross and John in 2003, is a tool developed to consider how people handle their emotions through two specific strategies: cognitive reappraisal (CR) and expressive suppression (ES). This questionnaire will be made up of 10 items ordered on a ranking from 1 (strongly disagree) to 7 (strongly agree). It includes four questions targeting expressive suppression and six questions focusing on cognitive reappraisal. Higher scores in each category reflect greater use of the respective ER strategy. the ERQ is known for its robust psychometric properties (62). In this study, the reliability of the ERQ was confirmed with Cronbach’s alpha scores of 0.73 for expressive suppression and 0.90 for cognitive reappraisal.

### 2.4 Data analyses

The study employed descriptive statistics to collection an overview of the participants’ socio-demographic characteristics and the scores of various study variables. To investigate the connections between AS and other variables, including mediators and outcomes, Pearson correlation analysis was conducted.

We applied a structural equation model (SEM) to test our hypothetical framework. Initially, the measurement models were assessed using the maximum likelihood (ML) approach, where all principal constructs were treated as latent variables. The suggested signs were described as follows: AS was represented by the combined scores of the three ASI-3 subscales; distress tolerance was reflected in the combined scores of the four DTS subscales; emotion regulation was denoted by the combined scores of the two ERQ subscales; and the total scores of health anxiety and negative consequences of illness denoted health anxiety. Ultimately, a SEM was designed to investigate the psychological pathways from AS to HA, considering sex and age as covariates for HA. The analysis was conducted using the ML method, and indirect effects were computed using a bootstrap technique with 5000 iterations. Statistical significance was determined at the 0.05 level if the bias-corrected bootstrap 95% confidence interval (CI) did not include zero (63). Based on the criteria proposed by Klin (2023), Chi-square index (χ2), comparative fit index (CFI; > 0.90), the goodness of fit index (GFI; < 0.95), the adjusted goodness of fit index (AGFI; < 0.90); in addition, values equal to or < 0.08 (values < 0.06 are more appropriate) for the RMSEA and SRMR suggest a good fit for the model (64). Statistical analyses were performed by utilizing the IBM SPSS (version 27.0) software package and the AMOS (version 24.0) software package.

## 3 Results

### 3.1 Descriptive statistics

**Table 1** provides an overview of the means, standard deviations, ranges, skewness, and kurtosis for the total and subscale scores of the SHAI, DTS, ASI-3, and ERQ-10. To assess the normality of these scores, normal Q-Q plots, histograms, and boxplots were visually inspected, and the kurtosis and skewness ratios were examined for each measure. The analysis indicated that all four measures followed an approximately normal distribution. There were no notable discrepancies between the mean and the five percent trimmed mean for each scale, suggesting that the data did not contain any significant outliers that could skew the results. Furthermore, the Cronbach’s alpha values for each scale exceeded the commonly accepted threshold of 0.70, implying strong internal consistency.

**Table 1:** Descriptive statistics for the ASI-3, DTS-15, ERQ-10 and SHAI.

Scale/subscale	N	Minimum	Maximum	Mean	SD	Skewness	Kurtosis
Distress Tolerance Scale (DTS)							
DTS – Total Scale	971	15	75	42.55	11.46	.06	-.46
DTS – Tolerance Subscale	971	3	15	9.47	3.19	-.40	-.58
DTS – Absorption Subscale	971	3	15	9.75	3.14	-.53	-.30
DTS - Appraisal Subscale	971	3	30	18.31	5.23	-.72	.30
DTS – Regulation Subscale	971	3	15	9.18	3.03	-.39	-.47
Anxiety Sensitivity Index-3 (ASI-3)							
ASI-3 – Total Scale	971	4	72	32.90	13.51	.28	.17
ASI-3 – Physical Subscale	971	0	24	10.54	4.69	.27	-.20
ASI-3 – Cognitive Subscale	971	0	24	11.73	5.24	.24	-.57
ASI-3 – Social Subscale	971	0	24	10.63	5.40	.17	-.48
Emotion Regulation Questionnaire (ERQ)							
Expressive suppression	971	4	28	15.74	4.61	-.20	-.65
Cognitive reappraisal	971	6	42	26.46	7.52	-.25	-.44
Short Health Anxiety Inventory (SHAI)							
SHAI – Health anxiety subscale	971	1	34	17.80	5.22	-.03	-.02
SHAI–Negative consequences of illness subscale	971	0	12	5.00	2.54	.17	-.13

### 3.2 Zero-order correlations

To explore the linkages among the study variables, Pearson correlation coefficients were calculated, and the results are presented in **Table 2**. The analysis revealed that AS had a statistically significant relationship with both DT and ER, and it was particularly positively correlated with HA.

**Table 2:** Pairwise correlations between the total and subscales of the DTS, ASI-3, ERQ-10 and SHAI.

Scale	1	2	3	4	5	6	7	8	9	10	11	12	13
1. DTS – Total Scale	–												
2. DTS – Tolerance Subscale	.85	–											
3. DTS – Absorption Subscale	.86	.79	–										
4. DTS - Appraisal Subscale	.89	.72	.73	–									
5. DTS – Regulation Subscale	.70	.47	.52	.52	–								
6. ASI-3 – Total Scale	-.40	-.32	-.35	-.38	-.29	–							
7. ASI-3 – Physical Subscale	-.34	-.27	-.30	-.31	-.23	.89	–						
8. ASI-3 – Cognitive Subscale	-.38	-.31	-.33	-.37	-.26	.88	.72	–					
9. ASI-3 – Social Subscale	-.33	-.26	-.29	-.33	-.26	.86	.66	.60	–				
10.ERQ – Expressive suppression Subscale	-.40	-.33	-.34	-.39	-.27	.44	.41	.41	.35	–			
11.ERQ – Cognitive reappraisal Subscale	.38	.28	.33	.36	.26	-.51	-.45	-.47	-.41	-.41	–		
12. SHAI – Total scale	-.46	-.38	-.38	-.40	-.31	.55	.50	.48	.46	.44	-.42	–	
13. SHAI – Health anxiety subscale	-.46	-.38	-.39	-.40	-.30	.50	.46	.44	.43	.41	-.39	.93	–
14. SHAI –Negative consequences of illness subscale	-.26	-.21	-.21	-.23	-.19	.40	.36	.34	.35	.33	-.30	.70	.41

DTS, Distress Tolerance Scale; ASI-3, Anxiety Sensitivity Index-3; ERQ, Emotion Regulation Questionnaire; SHAI, Short Health Anxiety Inventory; Each of these correlations was significant at the p ≤.01 level (two-tailed).

The relationships between the study’s exogenous, mediator, and endogenous variables, both positive and negative correlations, are shown in **Table 2**. The endogenous variable SHAI was moderately correlated with several variables: ASI-3 (r = .55, p <.001), DT (r = −.46, p <.001), ERQ-Expressive Suppression (ERQ-ES; r = .44, p <.001), and ERQ-Cognitive Reappraisal (ERQ-CR; r = −.42, p <.001). In contrast, the exogenous variable ASI-3 was negatively correlated with DT (r = −.40, p <.001), positively correlated with ERQ-ES (r = .44, p <.001), and negatively correlated with ERQ-CR (r = −.51, p <.001). Among the mediators, a significant negative relationship was discovered between DT and ERQ-ES (r = −0.40, p <.001), whereas DT and ERQ-CR exhibited a significant positive correlation (r = .38, p <.001).

### 3.3 SEM analyses

The study assessed the mediating role of DT and ER on the relationship between AS and HA. To evaluate the proposed model, one SEM analysis was performed. Since there was adequate evidence supporting the assumption of normality, the MLE method was employed (64).

**Figure 2:**
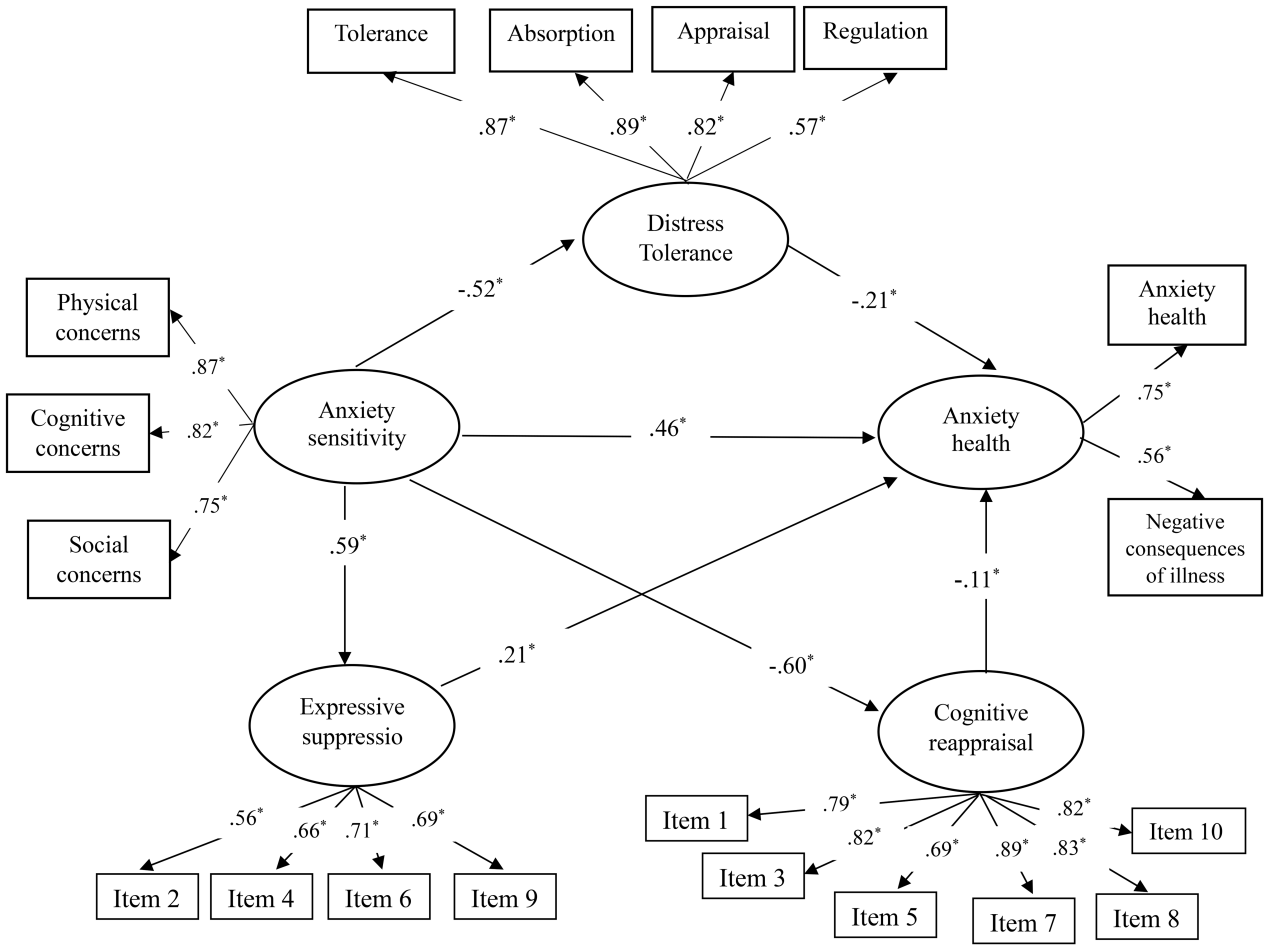
Final structural equation model. The symbol * indicates that the path coefficient is statistically significant, with p < 0.01.

The model’s fit was evaluated using several criteria: chi-square (χ²), normed chi-square index (χ²/df), comparative fit index (CFI), goodness of fit index (GFI), adjusted goodness of fit index (AGFI), root-mean-square error of approximation (RMSEA), and standardized root mean squared residual (SRMR). The results showed that the model fit well with the data (χ² = 434.421, df = 144, χ²/df = 3.01, p < 0.001, GFI = .953, AGFI = .938, RMSEA = .046, SRMR = .052). The normed chi-square index was 3.01, which is below the acceptable limit of 5 (58). The RMSEA value for the model was.046, which is below the standard cut-off of.05 (64). The CFI, GFI, and AGFI values were.90,.95, and.93, respectively, all of which exceed the recommended value of.90 (58), indicating that the model fit the data well. Subsequently, standardized path coefficients for all proposed paths were analyzed, and the findings are depicted in **Figure 2**.

**Table 3** provides an overview of the standardized direct, indirect, and total effects within the proposed model. Bootstrap analysis confirmed that the total indirect effect of AS on HA is statistically significant (β = .45, p <.01). Specifically, the indirect pathways from AS to HA through DT (β = .10, p <.01), ER-ES (β = .11, p <.01), and ER-CR (β = .06, p <.01) were significant. Moreover, significant direct effects of DT (β = -.20, p <.01), ER-ES (β = .21, p <.01), and ER-CR (β = -.11, p <.01) on HA were observed. The direct effect of AS on HA remained significant even after considering the indirect effects, indicating that DT, ER-ES, and ER-CR act as partial mediators. Please see **Table 3** for more information.

**Table 3:** Standardized direct, indirect and total estimates of the proposed model.

Model pathways	β	SE	95% CI	
Direct effect			LL	UL
Anxiety sensitivity → Health anxiety	.45^**^	.05	.34	.56
Anxiety sensitivity → Distress tolerance	-.52^**^	.03	-.59	-.45
Anxiety sensitivity → Expressive suppression	.59^**^	.03	.51	.66
Anxiety sensitivity → Cognitive reappraisal	-.60^**^	.03	-.66	-.52
Distress tolerance → Health anxiety	-.20^**^	.04	-.31	-.12
Expressive suppression → Health anxiety	.21^**^	.05	.11	.31
Cognitive reappraisal → Health anxiety	-.11^**^	.04	-.19	-.02
Indirect effect				
Anxiety sensitivity → Health anxiety (Total)	.28^**^	.04	.21	.37
Anxiety sensitivity → Distress tolerance → Health anxiety	.10^**^	.02	.06	.16
Anxiety sensitivity → Expressive suppression → Health anxiety	.11^**^	.03	.06	.18
Anxiety sensitivity → Cognitive reappraisal → Health anxiety	.06^**^	.02	.01	.11

**p<.01, n=917 (5.000 Bootstrap samples), β, standardized regression coefficient; SE, bootstrap standard error; CI, confidence interval; LL, lower limit; UL, upper limit.

## 4 Discussion

The primary objective of the study was to explore how emotion regulation (ER) and distress tolerance (DT) influence the connection between anxiety sensitivity (AS) and health anxiety (HA). We hypothesized that DT, cognitive reappraisal (CR) and expressive suppression (ES) would mediate the link between AS and HA. Our findings reveal a positive correlation between AS and HA, with DT and ER acting as mediators in this correlation.

We supposed that AS would be associated with HA, and our findings confirmed this prediction. We discovered a positive, significant, and moderate correlation between the Anxiety Sensitivity Index-3 and the short Health Anxiety Inventory. The study demonstrated that higher AS is linked to higher HA scores, aligning with previous research (19, 65–67). AS has been extensively discussed as a factor that may contribute to HA, playing a role in its development or exacerbation (68). AS may lead to hyper-vigilance towards bodily senses and increased distress when these senses occur, which are key components of the HA process (18). People with high AS are likely to monitor unexplained bodily sensations, such as a hot flash, to quickly detect and address potential threats (69, 70). Consequently, a person who thinks that unexplained bodily sensations indicate a medical catastrophe will constantly check their body for changes, quickly notice them, misinterpret them as threats, and then experience increased anxiety.

The current research’s findings show that AS related to ER. This finding is compatible with previous study that has shown connection between AS and ER (42–44). However, document suggests that in individuals with high AS, deficits in ER may contribute to the development of anxiety-related disorders (44). Pickett, Lodis, Parkhill, and Orcutt (2012) proposed a model that conceptualizes AS is a typical susceptibility characteristic for psychopathology, which may originate from broader susceptibility facets including personality traits (65). According to this approach, maladaptive ER, a more expansive risk element, may lead to an increased disposition towards AS. Operating this top-down model, targeting ER in therapy could potentially reduce AS or even preclude its onset (71). Therefore, improving ER skills may help alleviate individuals’ fear of anxiety symptoms (i.e., AS), thereby reducing the risk of developing anxiety-related disorders.

Our study found a significant negative connection between HA and CR (adaptive ER), and a significant positive relationship between HA and ES (Maladaptive ER). These results are consistent with previous research (14, 36, 38, 39, 72), as participants with high and moderate levels of HA showed low ER skills and vice versa. Consistent with these findings, research revealed that individuals with somatic symptom disorder utilized fewer adaptive ER procedures and more maladaptive ones compared to a healthy group. This suggests that adults with HA exhibit a reduced use of adaptive emotion regulation strategies (37). HA is linked to a negative mindset, where potential symptoms trigger thoughts like, “I am certain I will get sick”. This cognitive process can trigger a destructive cycle of affective conditions, manifesting as illness anxiety disorder, along with maladaptive cognitions and behaviors such as excessive information-seeking (73). Previous study has demonstrated a strong association between difficulties in ER and various anxiety-related behavioral and cognitive aspects (74). Challenges in ER may cause individuals to adopt ineffective coping strategies for health concerns, like seeking health information online instead of consulting healthcare professionals (75). Effective ER helps prevent excessive online health information seeking and is linked to reduced levels of HA.

Additionally, we anticipated that ER would be a mediator in the association between AS and HA. For individuals with high HA, regularly searching for medical information online about their symptoms is intended to offer short-term relief from recurring feelings and negative thoughts. Previous research indicates that difficulties in ER play a mediating role in this process (76). Poor ER leads to maladaptive coping strategies for HA, such as seeking health data online instead of consulting medical professionals (39). Thus, effective ER acts as a protective factor against HA and is also associated with low AS. Therefore, improving ER skills in individuals may be an indirect way to reduce HA.

Consistent with previous research demonstrating an inverse relationship between AS and DT (25, 28, 49, 77), the correlation investigations indicated that DT was negatively associated with AS. This implies that fear of anxiety-related symptoms (AS) increases as an individual’s capacity to tolerate distress diminishes. These results suggest that people with low DT are more prone to fear arousal-related sensations. It is possible that those encountering significant distress or anxiety develop a reduced tolerance for distress, which in turn makes them more sensitive to health symptoms.

The relation between DT and HA found in this study aligns with previous research in adults. Understanding this relationship involves analyzing the components that define DT: tolerance, appraisal, regulation, and absorption (17). Simons & Gaher

(2005) describe that individuals with lower tolerance for negative emotions experience heightened distress and sensitivity to anxiety (tolerance). They frequently underestimate their ability to manage anxiety indications (appraisal), which leads to the use of ineffective coping procedures, such as avoidance (regulation). Moreover, their intolerance of negative emotions disrupts their functioning because they are overly preoccupied with their distress (absorption). This kind of functional impairment is also characteristic of anxiety disorders (22, 28). The negative correlation between DT and HA identified in our research supports these findings. The results support previous research highlighting the transdiagnostic role of negative emotion tolerance in anxiety and HA (24, 27, 28, 47).

We examined the connection between DT and two ER strategies: CR (changing one’s thinking about a situation to change its emotional consequence) and ES (suppressing the outward expression of emotions). The findings showed a negative correlation between CR and DT, and a positive correlation between ES and DT. These findings are consistent with previous research (52, 56, 78), as our participants who demonstrated moderate and high levels of DT scored low in CR. As predicted, low DT was significantly related to greater use of ES. Research indicates that DT likely influences the ER strategies an individual adopts. For instance, someone with low DT may negatively interpret a situation because they perceive negative emotions as unbearable. This perception can drive them to use regulatory strategies like suppression, which provide immediate relief but may lead to long-term negative results (21). Supporting this theory, studies have found that individuals with low DT are more prone to conceal negative emotions and are more motivated to use ineffective ER strategies to manage negative emotional conditions (21, 22).

One explanation for this correlation is individuals who employ maladaptive regulation strategies, such as ES, are more likely to struggle with tolerating negative emotions. For example, individuals with high DT tend to assess their emotional experiences in a way that reduces the perceived need for ER. They possess a greater sense of confidence in their ability to manage distressing feelings, view negative emotions as non-threatening, and do not feel an immediate urge to avoid or reduce these emotions (22, 56). Consequently, they are more likely to accept their emotions without attempting to alter them (56). Another explanation is that individuals with higher DT often interpret emotional experiences (reappraisal) in a way that reduces their perceived need for ER. They believe they can manage distress effectively, view negative emotions as non-threatening, and do not feel a strong need to avoid or reduce these emotions (22). This perspective aligns with the idea that emotions do not require control, leading to less frequent use of active ER strategies (56). In our sample, this was evident from the association between higher DT and increased CR (reappraisal). Further research into how beliefs about emotion affect the association between DT and ER behaviors could provide additional insights into these phenomena.

Recent developments within the metacognitive model of emotion dysregulation (79) provide additional insights into the relationship between DT and ER. According to this model, distress internal states (e.g., negative thoughts or emotions) may activate dysfunctional metacognitive beliefs, which can lead to greater difficulties in ER, either directly or via maladaptive forms of mental control (79). Evidence from non-clinical and clinical samples, including individuals with affective disorders, eating disorders, and substance use disorders, supports the notion that these dysfunctional metacognitive processes exacerbate emotion dysregulation (80–82). For example, individuals who perceive their negative emotions as uncontrollable or catastrophic are more likely to engage in maladaptive ER strategies, such as avoidance or suppression, further reducing their DT (81, 82). Incorporating these perspectives into the current findings highlights the importance of targeting both metacognitive beliefs and ER strategies in interventions aimed at improving DT and reducing HA. Future research should explore how these metacognitive factors interact with DT and ER to influence HA, potentially offering new avenues for therapeutic approaches.

Furthermore, our study investigates the effect of mediation of DT and ER on the connection between AS and HA. The outcomes of bootstrapped parallel mediation studies showed that ER and DT partially mediated the association between AS and HA symptoms. This suggests that individuals with a higher capacity for emotional tolerance experience less emotional distress, which enables them handle negative automatic thoughts and prevent HA.

### 4.1 Limitations and directions for future studies

The findings of our study should be interpreted with caution due to several important limitations. These constraints are essential to consider when evaluating the results of this initial research. Firstly, the cross-sectional format of this study prohibits for conclusions about causality or the determination of temporal relationships. Although our findings suggest that DT and ER mediate the relationship between AS and HA, these conclusions should be considered exploratory and correlational rather than definitive. Longitudinal studies are needed to examine the directionality and potential causal pathways between AS, ER, DT, and HA over time. Without such evidence, we cannot definitively claim that improving DT or ER will directly reduce HA, or that these mediators are the underlying mechanisms of the relationship between AS and HA. Secondly, the sample composition must be acknowledged as a limitation. Our participants were not clinical subjects, and this limits the generalizability of the findings to populations with diagnosed health anxiety-related disorders, including illness anxiety disorder. The results may not apply to individuals with more severe manifestations of HA. Future studies should replicate this research with clinical populations to better understand how ER and DT operate in individuals with more pronounced HA symptoms. Thirdly, the use of convenience and snowball sampling methods in this study makes it difficult to ascertain whether the sample accurately represents the entire population of Iran. Future studies should consider using larger sample sizes and exploring the relationships between variables in diverse populations. Fourthly, the use of web-based surveys limits the ability to create random samples, as participants need internet access, leading to potential self-selection biases. It is recommended that this research be replicated on a larger national scale and that qualitative methodologies be used to uncover cultural nuances that quantitative methods cannot capture. Fifthly, although there are known disparities among ethnic groups in ER (62), this study did not control for potential cultural variances. In addition, there is a shortage of specific demographic data such as ethnicity, race, and socioeconomic status necessitates cautious interpretation of our findings. A further limitation is the reliance on self-report measures, which might potentially create common method variance. Future research could profit from adopting multimethod approaches, such as behavioral indicates of ER and DT.

On the clinical level, these findings show that those who fail to tolerate unpleasant emotions are at greater risk to develop different kinds of psychopathology related with emotion dysregulation, such as illness anxiety disorder. Our results indicate that treatments aimed at reducing HA symptoms by focusing on DT and ER could be particularly effective. Broadly targeting DT and ER may help lower HA levels. Understanding the role of DT as a mediator can assist physicians recognize those patients who will probably to benefit the most from specific therapies and establish suitable treatment suggestions. For example, people who have low DT levels might benefit more from Dialectical Behavior Therapy (DBT), which is specifically designed to enhance DT skills. DBT, a form of cognitive-behavioral therapy, incorporates Instruction in skills and functioning behavioral assessment alongside ideas of dialectical philosophy, mindfulness and styles of communication (83). DBT has been shown to improve ER in individuals with various anxiety and mood disorders (84). Since DBT targets ER, it may be an effective treatment for HA. Although no studies have yet employed DBT specifically for HA, research combining ER treatments with DBT has shown positive outcomes for individuals with HA. Additionally, DBT can educate persons who employ inefficient coping mechanisms in response to an unable to handle unpleasant negative emotions to find more effective strategies for addressing negative impact.

Our findings emphasize the importance of targeting both DT and ER in therapeutic interventions for HA. Cognitive-behavioral therapy (CBT) has been shown to be effective in enhancing DT by equipping individuals with skills to endure and manage distressing emotions (85). Additionally, metacognitive therapy (MCT) offers a promising approach to addressing difficulties in ER by focusing on modifying dysfunctional metacognitive beliefs (86). MCT aims to reduce the reliance on maladaptive forms of mental control and promote adaptive strategies for managing emotional distress. Incorporating these therapeutic approaches may improve outcomes for individuals experiencing health anxiety by addressing both the underlying mechanisms of DT and ER. These insights contribute to a more comprehensive understanding of the relationship between AS and HA and highlight the potential for integrative therapeutic approaches that address the interplay of DT, ER, and metacognitive processes.

## 5 Conclusions

As the first study to examine the role of DT and ER in explaining the link between AS and HA in an Iranian population, our results showed a strong correlation between AS and HA, consistent with existing adult literature. These findings show that AS is a key risk factor for HA, with DT and ER playing significant roles in this relationship. Future longitudinal studies are needed to affirm our findings, and AS and its related mechanisms should be considered in HA treatment. Although our study adds valuable insights into the understanding of HA, it represents only a portion of the knowledge necessary to develop a developmental, evidence-based model of HA. Understanding the causes of HA better will help in developing evidence-based evaluations and interventions for controlling HA.

## Data Availability

The raw data supporting the conclusions of this article will be made available by the authors, without undue reservation.
